# Circulating Tumor Cells as a Biomarker of Response to Treatment in Patient-Derived Xenograft Mouse Models of Pancreatic Adenocarcinoma

**DOI:** 10.1371/journal.pone.0089474

**Published:** 2014-02-19

**Authors:** Robert J. Torphy, Christopher J. Tignanelli, Joyce W. Kamande, Richard A. Moffitt, Silvia G. Herrera Loeza, Steven A. Soper, Jen Jen Yeh

**Affiliations:** 1 University of North Carolina School of Medicine, Chapel Hill, North Carolina, United States of America; 2 Department of Surgery, University of North Carolina, Chapel Hill, North Carolina, United States of America; 3 Department of Chemistry, Louisiana State University, Baton Rouge, Louisiana, United States of America; 4 Lineberger Comprehensive Cancer Center, University of North Carolina, Chapel Hill, North Carolina, United States of America; 5 Department of Biomedical Engineering, University of North Carolina, Chapel Hill, North Carolina, United States of America; 6 Department of Chemistry, University of North Carolina, Chapel Hill, North Carolina, United States of America; 7 Department of Pharmacology, University of North Carolina, Chapel Hill, North Carolina, United States of America; University of Kentucky College of Medicine, United States of America

## Abstract

Circulating tumor cells (CTCs) are cells shed from solid tumors into circulation and have been shown to be prognostic in the setting of metastatic disease. These cells are obtained through a routine blood draw and may serve as an easily accessible marker for monitoring treatment effectiveness. Because of the rapid progression of pancreatic ductal adenocarcinoma (PDAC), early insight into treatment effectiveness may allow for necessary and timely changes in treatment regimens. The objective of this study was to evaluate CTC burden as a biomarker of response to treatment with a oral phosphatidylinositol-3-kinase inhibitor, BKM120, in patient-derived xenograft (PDX) mouse models of PDAC. PDX mice were randomized to receive vehicle or BKM120 treatment for 28 days and CTCs were enumerated from whole blood before and after treatment using a microfluidic chip that selected for EpCAM (epithelial cell adhesion molecule) positive cells. This microfluidic device allowed for the release of captured CTCs and enumeration of these cells via their electrical impedance signatures. Median CTC counts significantly decreased in the BKM120 group from pre- to post-treatment (26.61 to 2.21 CTCs/250 µL, p = 0.0207) while no significant change was observed in the vehicle group (23.26 to 11.89 CTCs/250 µL, p = 0.8081). This reduction in CTC burden in the treatment group correlated with tumor growth inhibition indicating CTC burden is a promising biomarker of response to treatment in preclinical models. Mutant enriched sequencing of isolated CTCs confirmed that they harbored *KRAS* G12V mutations, identical to the matched tumors. In the long-term, PDX mice are a useful preclinical model for furthering our understanding of CTCs. Clinically, mutational analysis of CTCs and serial monitoring of CTC burden may be used as a minimally invasive approach to predict and monitor treatment response to guide therapeutic regimens.

## Introduction

Tumor cells that are present in peripheral circulation, or circulating tumor cells (CTCs), have been isolated from blood samples of patient's with many solid cancers. These cells are an attractive target for staging and monitoring treatment effectiveness because they are obtained noninvasively through a routine blood draw and therefore can be measured serially throughout the course of treatment. CTC burden has been shown to be predictive of survival in metastatic breast, colorectal, prostate and lung cancers [1]–[5]. CTCs have been isolated from patients with pancreatic ductal adenocarcinoma (PDAC) but investigation of their clinical utility has proven less successful than in other epithelial cancers [6].

PDAC is a devastating disease characterised by early and aggressive metastasis with a five year survival rate of <5% [7]. Depending upon the extent of disease at diagnosis, the current standard of care includes surgical resection, radiation therapy, and chemotherapy with gemcitabine. Unfortunately >85% of patients with PDAC present with disseminated or inoperable disease and are not candidates for curative surgery [8]. New chemotherapeutics and clinical approaches for treating PDAC are needed. The Ras pathway is a highly sought after therapeutic target due to the high frequency of *KRAS* mutations found in up to 95% of PDAC [9]. Despite much effort, no anti-Ras therapies have been successful. Currently, promising therapies focus on targeting downstream effectors of Ras such as the Raf-MEK-ERK mitogen-activated protein kinase (MAPK) and phosphatidylinositol-3-kinase (PI3K)-AKT signaling pathways [10]. PI3K is an attractive therapuetic target as it is one of the main Ras effector signaling pathways, is involved in tumor growth and maintenance, and has also been reported to be mutated in pancreatic cancers [11], [12], [13]. Ras is known to directly interact with the p110α catalytic subunit of PI3K and this interaction is imporant for Ras-driven tumor formation [14], [15]. Given this, therapeutically targeting the p110α catalytic subunit may be effective in tumors harboring either *KRAS* or *PI3KCA* mutations. BKM120 is an oral pan-class 1 PI3K inhibitor that inactivates the p110α subunit and is currently in Phase I–III clinical trials [16]. To date, the effectiveness of BKM120 in PDAC is unknown. However, *in vitro* studies of various cancer cell lines have shown that BKM120 decreases phosporylated-Akt (p-Akt) levels, inhibits signaling pathways downstream of PI3K and p-Akt, and induces apoptosis [17].

Patient-derived xenografts (PDX) are known to be an excellent preclinical model for oncology drug development and biomarker discovery. PDX mouse models are created by engrafting surgically resected patient tumor samples subcutaneously in immunocompromised mice. PDX tumors can be passaged over time and expanded into subseqent generations of mice while still maintaining the tumor architecture, genetic heterogeneity, and mutational profile as the primary tumor [18], [19]. PDX more accurately model the primary tumor than traditional cell-line derived xenografts that are more genetically homogenous and have adapted to growth *ex-vivo*. However, studies of CTCs in preclinical models has been limited to cell–line derived xenografts or genetically engineered mice to date [20], [21].

To assess CTC burden in our preclinical model, a novel microfluidic chip that selects for EpCAM positive cells from as little as 250 µL of whole blood was used. This microfluidic chip allows for captured CTCs to be released from the capture surface for enumeration using unique electrical impedance signatures of CTCs as they pass through two platinum (Pt) electrodes. The electrical impedence enumeration has high sensitivity and specificity for CTCs based on their size and electrical properties compared to any contaminating leukocytes or red blood cells [22], [23]. In addition, the microfluidic chip has been shown to demonstrate high clinical sensitivity, even for PDAC CTCs when using EpCAM positive selection, providing the ability to collect sufficient numbers of CTCs to monitor drug response in PDX models using non-terminal bleeds [24].

As more personalized therapies like BKM120 become available to treat PDAC, clinicians will have the flexibility to personalize therapies to achieve optimal response. Biomarkers such as CTCs may guide therapy in several ways. First, early insight into treatment effectiveness by monitoring CTC burden may help guide changes in therapy. Second, CTC mutational status, which may or may not be entirely the same as the primary tumor, may guide therapy selection. Third, profiling changes in CTC gene expression may predict treatment resistance [20]. Because PDAC has such a rapid progression and poor prognosis, early evaluation of treatment effectiveness and early intervention in these patients is of great importance for prolonging survival and improving quality of life. Therefore the objective of this study was to first evaluate CTCs in patient derived xenograft (PDX) mouse models of PDAC and secondly to evaluate CTC burden as a biomarker for response to targeted treatment with BKM20.

## Materials and Methods

### PDX Cohort Expansion

PDAC tumor tissue from a de-identified patient was obtained from the University of North Carolina Institutional Review Board (IRB) approved tissue procurement facility after IRB approval. Tumor tissue was engrafted subcutaneously into the flanks of NSG/NOD mice, expanded, and passed over time. All animal experiments were carried out ethically under protocols approved by the University of North Carolina Institutional Animal Care and Use Committee (protocol # 12–314).

### Treatment

Treatment was begun when tumors reached a median size of 240 mm^3^ (range  = 121–487 mm^3^) in the expansion cohort. Mice were randomized and dosed with either vehicle or BKM120 (40 mg/kg) by oral gavage (o.g.) once daily for 28 days. BKM120 was suspended in 1 volume NMP (1-methyl-2-pyrrolidone): 9 volumes PEG300 with a total application volume of 10 mL/kg [25]. Mice in the vehicle group were treated with NMP/PEG300 vehicle with a total application volume of 10 mL/kg. Caliper measurements were performed twice weekly and tumor volume was calculated using the formula [½ (width × length^2^)]. On treatment day 28, the mice were euthanized and the tumors harvested and flash frozen for subsequent analysis.

### Microfluidic Chip Fabrication

**Figure 1:**
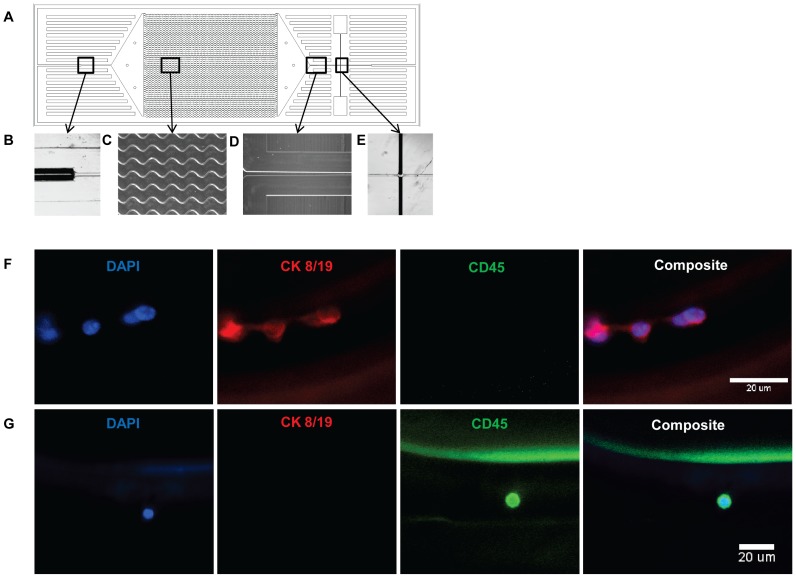
CTC isolation using a microfluidic chip. (A) Design of the CTC microfluidic chip with sinusoidally shaped capture channels and brightfield images of (B) the capillary tube inserted into the on-chip entry channel where whole blood enters the microfluidic chip, (C) sinusoidally shaped capture channels where anti-human EpCAM antibodies are immobilized for CTC capture, (D) exit channel, and (E) impedance sensor with two Pt electrodes located adjacent to the exit channel to detect released CTCs. (F) Cells captured from whole blood of PDX-tumor bearing mice visualized directly on the microfluidic chip following immunostaining with DAPI (blue), human cytokeratin 8/19 (CK, red), and mouse CD45 (green). The staining pattern of DAPI-positive, human CK-positive, and mouse CD45-negative is characteristic of human CTCs. (G) A rare contaminating mouse leukocyte bound non-specifically to the microfluidic chip stained DAPI-positive, human CK-negative, and mouse CD45-positive. Contaminating leukocytes were excluded from enumeration due to the high specificity of the electrical impedance detector for cancer cells.

The microfluidic chip that was used to capture CTCs in this study consists of 51 sinusoidally shaped channels, 30 µm wide and 150 µm deep, that has been optimized to isolate CTCs from small volumes of whole blood (Figure 1, A–E) [22]. Microfluidic chips were hot-embossed into PMMA as previously described [23]. Following ultraviolet modification of the microfluidic channels, cover slips were annealed to the chips at 98°C for 25 min. Monoclonal anti-human EpCAM antibodies (R&D Systems) were immobilized to the microfluidic channels in a two step process. First, a solution of 200 mg/mL of EDC (1-ethyl-3-[3-dimethylaminopropyl] carbodiimide hydrochloride) (ThermoScientific) and 50 mg/mL of NHS (*N*-hydroxysuccinimide) (ThermoScientific) in 100 mM MES (2-[4-morpholino]-ethane sulfonic acid) (pH = 4.83) was infused into the microfluidic chip and incubated at room temperature for 10 min. Following incubation, the EDC/NHS solution was replaced with 0.5 mg/mL monoclonal anti-human EpCAM antibodies in PBS (pH = 7.4) and allowed to react overnight at 4°C. Prior to CTC capture, each microfluidic chip was rinsed with a solution of phosphate-buffered saline (PBS) to remove any nonspecifically bound antibody at a flow rate of 55 µL/min.

### CTC Isolation and Enumeration

On day 0 prior to treatment with BKM120 or vehicle, blood was collected from each mouse via a submandibular bleed. Terminal bleeds were performed via cardiac puncture on treatment day 28. Blood was collected in ethylenediaminetetraacetic acid (EDTA) tubes and processed within five hours of collection. To isolate CTCs from whole blood, blood was introduced into the microfluidic chips at a flow rate of 27.5 µL/min. Processed blood volumes for the day 0 samples ranged from 180–380 µL (mean  = 255 µL); volumes of the day 28 samples ranged from 650–1000 µL (mean  = 929 µL) The chips were then washed with 0.2 mL of PBS at a flow rate of 55 µL/min. Captured cells were released from the microfluidic chip with 0.25% trypsin for enumeration using electrical impedance signatures of CTCs. Impedance measurements were obtained using previously described, home-built circuitry, capable of detecting single tumor cells as they travel through the detection electrodes with high sensitivity and specificity [22]. Data were collected using NI-USB-6009 (National Instruments) data acquisition board and software written in LabView (National Instruments). After Gaussian filtering, peaks in the data trace that were greater than 10 times the standard deviation of the background noise were considered significant and counted as CTCs using a custom user interface in MATLAB.

### Immunofluorescence

Staining was performed directly on the microfluidic chip following CTC isolation from whole blood. 0.25% Bovine Serum Albumin (BSA) in PBS buffer (pH 7.4) was infused through the microfluidic chip for 10 min at a flow rate of 20 µL/min. Next, 100 µL of 0.0025 mg/mL of anti-mouse CD45-FITC (HI30 clone; eBiosciences) was infused at a flow rate of 10 µL/min and incubated at 4°C for 1 hour followed by a 5 min wash of 0.02% TX-100/PBS at 20 µL/min. For fixation and permeabilization, 2–4% paraformaldehyde in PBS buffer was infused at a flow rate of 15 µL/min for 10 min followed by 0.1% TX-100 in PBS buffer at a flow rate of 15 µL/min for 10 min. Next, 100 µl of a panel of 0.01 mg/mL anti-Cytokeratins 8, 19 labeled with Texas Red (eBiosciences) was infused through the device at 10 µL/min and incubated at 4°C for ∼1 hour, followed with a 10 min wash of 0.25% BSA in PBS at 20 µL/min. For nuclear staining, 0.001 mg/mL DAPI (Thermo Pierce Technologies) in PBS was infused at a flow rate of 15 µL/min for 7 min followed by a final 15 min wash with 0.25% BSA in PBS.

The CTC microfluidic chip was manually scanned on an inverted Olympus 1×71 microscope (Center Valley, PA) using 10x, 20x, 40x, and 60x dry objectives equipped with a high resolution (1344×1024) CCD camera (Hamamatsu ORCA-03G) and a mercury arc lamp as an illumination source. Images were collected and analyzed using Metamorph imaging software (Olympus).

### DNA Isolation

After CTCs were enumerated via electric impedance measurements, they were collected in 100 uL Fetal Bovine Serum (FBS) and stored at −80°C. At the time of DNA isolation, samples were thawed and centrifuged at 10,000 RPM for 2 min to sequester cells at the bottom of the collecting tube. All but 100 µL of supernatant was removed and cells were resuspended in 350 µL of Buffer RLT Plus (Qiagen). The sample was then vortexed for 30 seconds and CTC DNA was isolated using the AllPrep DNA Kit (Qiagen) or the QIAamp DNA Micro Kit (Qiagen). Tumor DNA was isolated using the AllPrep DNA Kit.

### Mutational Analysis of Tumor DNA

Direct sequencing mutational analysis was performed for *PI3K* codons 542, 545, and 1047, and *KRAS* codons 12 and 13 using polymerase chain reaction (PCR) amplification followed by pyrosequencing (Pyromark MD, Qiagen). For sequencing of *PI3K* codons 542, 545, and 1047, PCR of exons 9 and 20 was performed using the following primers: exon 9, 5′–/BioTEG/ATTTCTACACGAGATCCTCTCTCT3′ and 5′–CCATTTTAGCACTTACCTGTGAC–3′; exon 20, 5′– TGAGCAAGAGGCTTTGGAGTAT–3′ and 5′–/BioTEG/TGCTGTTTAATTGTGTGGAAGATC–3′. PCR amplification was done for 45 cycles with an annealing temperature of 62°C. PCR products were analyzed by pyrosequencing using the internal primers: codon 542, 5′–TTCTCCTGCTCAGTGAT– 3′; codon 545, 5′– TAGAAAATCTTTCTCCTG–3′; codon 1047, 5′–GAAACAAATGAATGATGC–3′.

For sequencing of *KRAS* codons 12 and 13, PCR of exon 2 was performed using the primers: 5′–CGATGGAGGAGTTTGTAAATGAA–3′and 5′–/BioTEG/TTCGTCC ACAAAATGATTCTGA–3′. PCR amplification was done for 55 cycles with an annealing temperature of 68°C. PCR products were analyzed by pyrosequencing using the internal primer 5′–AAACTTGTGGTAGTTGGA–3′.

### KRAS Mutational Analysis of CTCs

MES protocols for *KRAS* codon 12 have previously been defined [26], [27]. In brief, the first PCR was performed using 1× Immomix buffer with 4 µL of CTC DNA sample, and 10 µM of each primer. The primers used were: 5′– ACTGAATATAAACTTGTGGTAGTTGGACCT–3′ and 5′– TCAAAGAATGGTCCTGGACC– 3′. Touchdown PCR was used for the first round of amplification to selectively amplify the human *KRAS* gene. After an initial 15 min at 94°C, cycling conditions were 60 s at 94°C, 40 s at 62°C, 40 s at 72°C. For the next 10 cycles the annealing temperature was decreased by 0.5°C for each cycle until a final annealing temperature of 57.5°C was reached. Products were subjected to an additional 30 cycles (60 s at 94°C, 40 s at 57.5°C, 40 s at 72°C) of PCR. PCR products were then purified, quantified, and subjected to restriction digest by the addition of BstNI and NEBuffer 3.1(New England Biolabs) for 16 hours at 60°C. Restriction digested products were then diluted to a final concentration of 1–2 ng of DNA/µL.

The second round of PCR was done using the primers 5′–/BIOTEG/ACTGAATATAAACTTGTGGTAGTTGGACCT– 3′ and 5′– TAATATGCATATTAAAACAAGATTTACCTC– 3′, for 40 cycles (60 s at 94°C, 40 s at 54°C, 40 s at 72°C). PCR products were analyzed using pyrosequencing (Pyromark MD, Qiagen) using the internal primer 5′– CGTCAAGGCACTCTTGCC–3′.

### Statistical Analysis

Statistical analysis was performed to compare CTCs enumerated from non-tumor bearing mice to PDX-tumor bearing mice, change in CTC burden with treatment, and change in tumor volume with treatment. CTC data was analyzed using a non-parametric test (Wilcoxon-Mann-Whitney test) because the data had a non-Gaussian distribution. Tumor volume data had a Gaussian distribution and was analyzed using a parametric test (unpaired t-test). Statistical analysis of absolute CTC burden vs. absolute tumor volume and change in CTC burden vs. change in tumor volume was performed using Pearson correlation.

## Results and Discussion

### Isolation and Enumeration of CTCs from PDAC PDX Mice

Whole blood was introduced directly into the microfluidic chip without pre-processing and EpCAM positive cells were selected for by interaction with anti-human EpCAM immobilized to the walls of the microfluidic channels. To verify that CTCs were captured on the microfluidic chip, immunofluorescence staining was performed directly within the microfluidic chip. One accepted method for identification of CTCs via immunofluorescence is nuclear staining (DAPI) positive, epithelial cytokeratin (CK) positive, and leukocyte CD45 negative [28]. Captured cells stained DAPI-positive, human CK 8/19-positive, and mouse CD45-negative, confirming their identity as CTCs (Figure 1, F). Non-specifically bound leukocytes were also found within the microfluidic chip, staining DAPI-positive, human CK 8/19-negative, and mouse CD45-positive (Figure 1, G). From seven samples analyzed by immunofluorescence staining, the purity of captured CTCs to contaminating leukocytes was 89% (range 62–100%), with an average count of 1.5 leukocytes per 250 µL of processed blood (range 0–5 leukocytes/250 µL).

Unfortunately, immunofluorescence staining within the microfluidic chip and manually locating and identifying CTCs is time intensive. To automate the enumeration process, captured cells were released from the capture surface and enumerated based on impedance measurement which is capable of discriminating cancer cells from contaminating leukocytes based on cell size as the cells passed between two Pt electrodes. Previous investigation into the specificity of using impedance for enumerating cancer cells showed that there is only minimal misclassification (∼15%) when this method is used to identify cancer cells vs. leukocytes. This misclassification rate correlated with the overlap in the size distribution of these two cell populations [24]. Enumeration of CTCs by impedance measurements allowed for a fully automated enumeration process.

**Figure 2:**
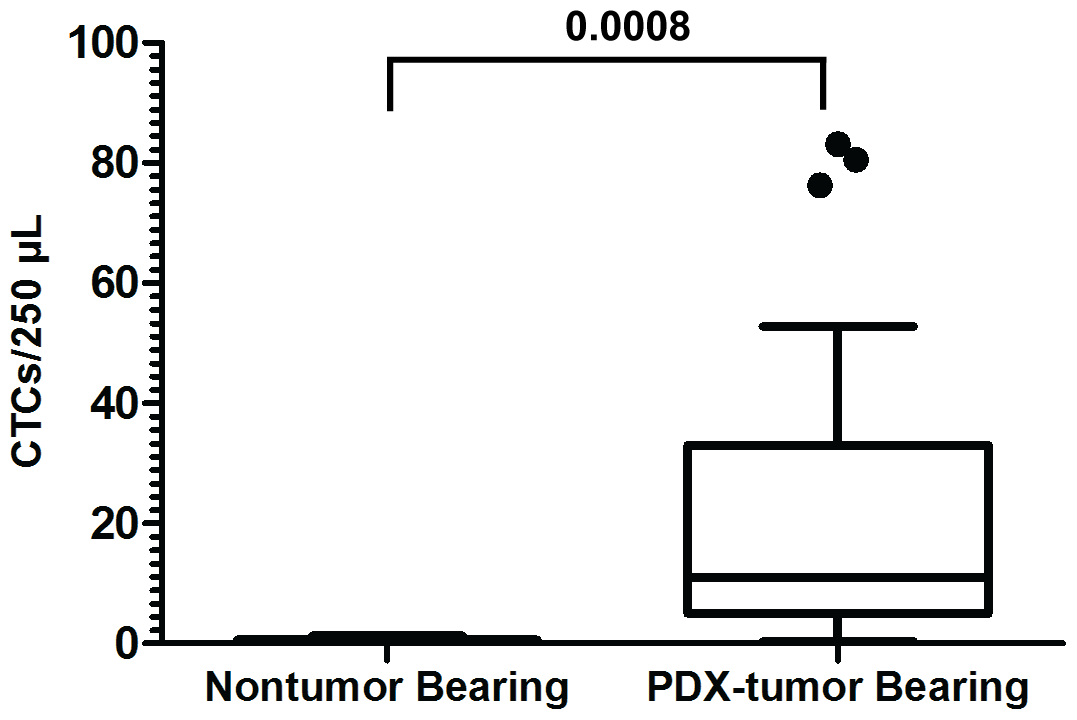
CTC enumeration from PDX-tumor bearing mice. CTCs captured and enumerated from whole blood of non-tumor and tumor bearing PDX mice. Five non-tumor bearing mice were analyzed for their CTC level (median  = 0 CTCs/250 µL, range  = 0–1 CTCs/250 µL), while CTCs were enumerated from 31 of 31 PDAC PDX mice (median  = 11 CTCs/250 µL, range  = 1–83 CTCs/250 µL) (p = 0.0008, Wilcoxon).

Previous work from the Haber laboratory using a similar CTC microfluidic chip showed that CTCs, defined by a staining pattern of CK-positive, EpCAM-positive, and CD45-negative, were found at a very low level in blood from healthy subjects (median  = 1.2 CTCs/100 µL, range  = 0–2.2 CTCs/100 µL) [20]. The capability our microfluidic chip to accurately detect and enumerate CTCs by impedance as well as the absence of CTC burden in healthy controls was confirmed in this study through the processing and enumeration of CTCs from whole blood from non-tumor bearing PDX mice and PDAC PDX mice. Zero CTCs were enumerated from four out of five non-tumor bearing PDX mice (median  = 0 CTCs/250 µL, range  = 0–1 CTCs/250 µL), while CTCs were enumerated from 31 of 31 PDAC PDX mice (median  = 11 CTCs/250 µL, range  = 1–83 CTCs/250 µL) (p = 0.0008, Wilcoxon) (Figure 2). While 1 non-tumor bearing PDX sample had a positive reading, only one cell was responsible for this false positive reading. Our immunofluorescence staining results showing that leukocyte contamination is found on the microfluidic chip with an average count of 1.5 leukocytes per 250 µL of processed blood (range 0–5 leukocytes/250 µL), coupled with the ∼15% misclassification rate between CTCs and leukocytes using impedance enumeration, suggests that we expect an average false positive rate of only 0.225 cells per 250 µL of processed blood (range 0–0.75 cells/250 µL). The presence of only 1 out of 5 non-tumor bearing mice with a false positive reading of one cell is in concordance with these expectations. Therefore, impedance is an accurate method for detection of CTCs given the high purity of CTCs captured on the microfluidic chip (89%), the low misclassification rate (∼15%) between cancer cells and leukocytes, and the significantly higher burden of CTCs enumerated from tumor-bearing PDX mice.

### CTC Response to BKM120 Treatment

**Figure 3:**
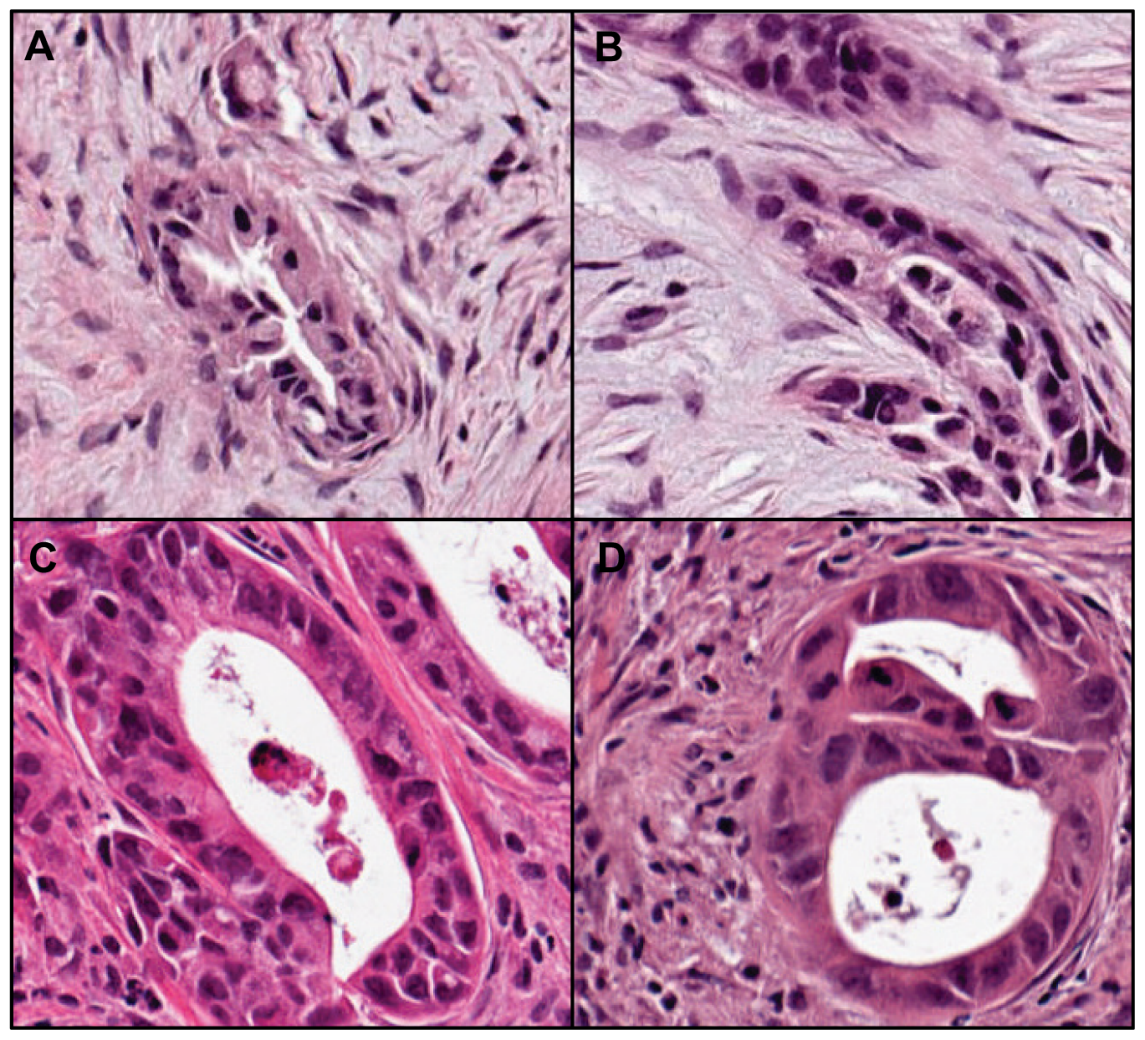
Patient and PDX tumor. H&E staining of the de-identified patient tumor (A, B) and corresponding PDX tumor (C, D). Both the patient and PDX tumors are consistent with PDAC with desmoplastic stroma (20× magnification).

**Figure 4:**
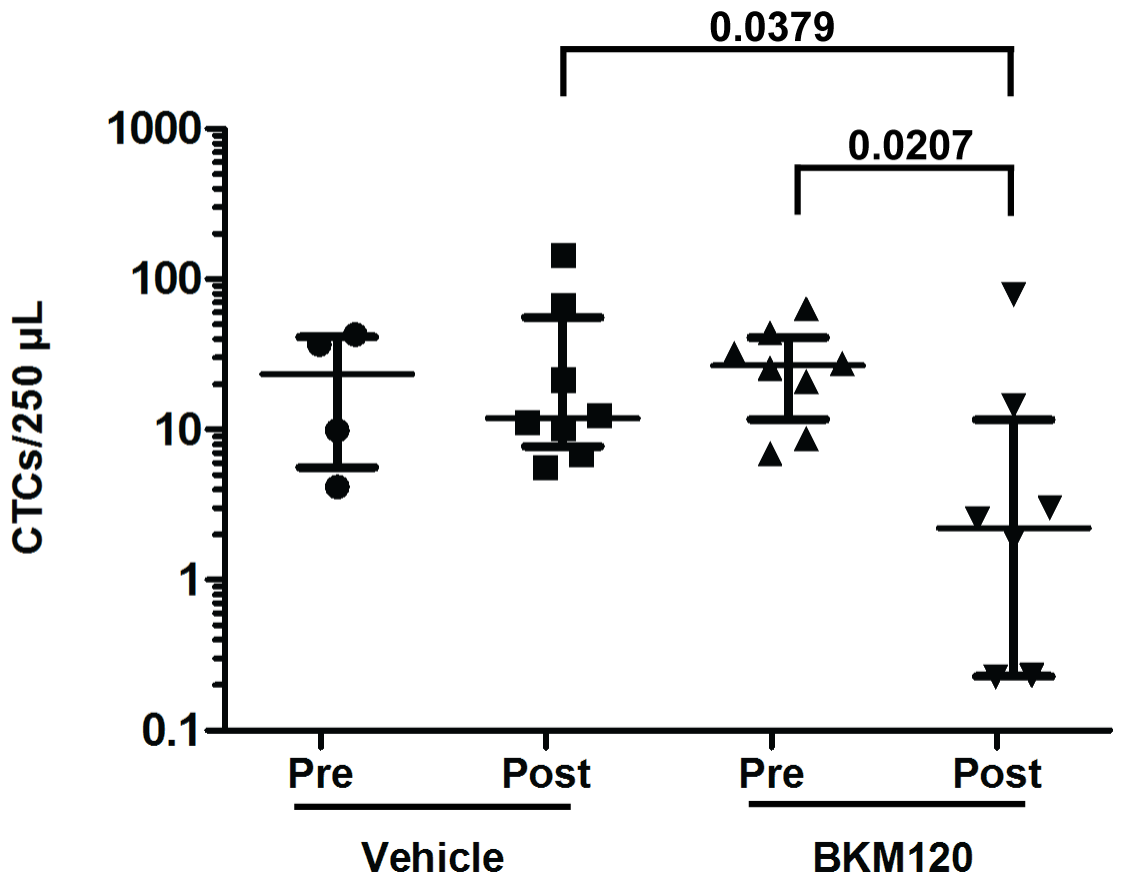
Response of CTC burden to BKM120 treatment. PDAC PDX mice were treated with vehicle or BKM120 for 28 days. CTCs were enumerated from whole blood on day 0 prior to the first treatment and on day 28 after the last treatment. CTC counts significantly decreased in the BKM120 group from pre- to post-treatment (pre-treatment, pre: median  = 26.61 CTCs/250 µL, range  = 7–63 CTCs/250 µL, n = 8; post-treatment, post: median  = 2.21 CTCs/250 µL, range  = 0–79 CTCs/250 µL, n = 8; p = 0.0207, Wilcoxon) while no significant change was observed in the vehicle group (pre: median  = 23.26 CTCs/250 µL, range  = 4–43 CTCs/250 µL, n = 4; post: median  = 11.89 CTCs/250 µL, range  = 6–146 CTCs/250 µL, n = 8; p = 0.8081, Wilcoxon) One post BKM120 treatment sample had no detectable CTCs and is not plotted on scale.

A cohort of PDX mice was expanded from a de-identified PDAC patient tumor. Hematoxylin and eosin staining showed that the PDX tumor tissue maintained a similar histological architecture as the primary patient tumor with marked desmoplastic stroma (Figure 3). Mice were randomized to receive vehicle or BKM120 for 28 days. Blood was collected from PDX mice for CTC enumeration on day 0 prior to the first treatment with BKM120 or vehicle and again on day 28. On day 0, there was no significant difference in baseline CTC counts between the BKM120 group and vehicle group (p = 0.8081). Median CTC burden significantly decreased in the BKM120 group from pre- to post-treatment, with one mouse having no detectable CTCs at post-treatment (pre-treatment, pre: median  = 26.61 CTCs/250 µL, range  = 7–63 CTCs/250 µL, n = 8; post-treatment, post: median  = 2.21 CTCs/250 µL, range  = 0–79 CTCs/250 µL, n = 8; p = 0.0207, Wilcoxon) while no significant change was observed in the vehicle group (pre: median  = 23.26 CTCs/250 µL, range  = 4–43 CTCs/250 µL, n = 4; post: median  = 11.89 CTCs/250 µL, range  = 6–146 CTCs/250 µL, n = 8; p = 0.8081, Wilcoxon) (Figure 4). The reduction in CTC burden that was observed in the BKM120 treatment group suggests that changes in CTC burden may be used to non-invasively monitor response to therapy in patients with pancreatic cancer.

### Tumor Response to BKM120 Treatment

**Figure 5:**
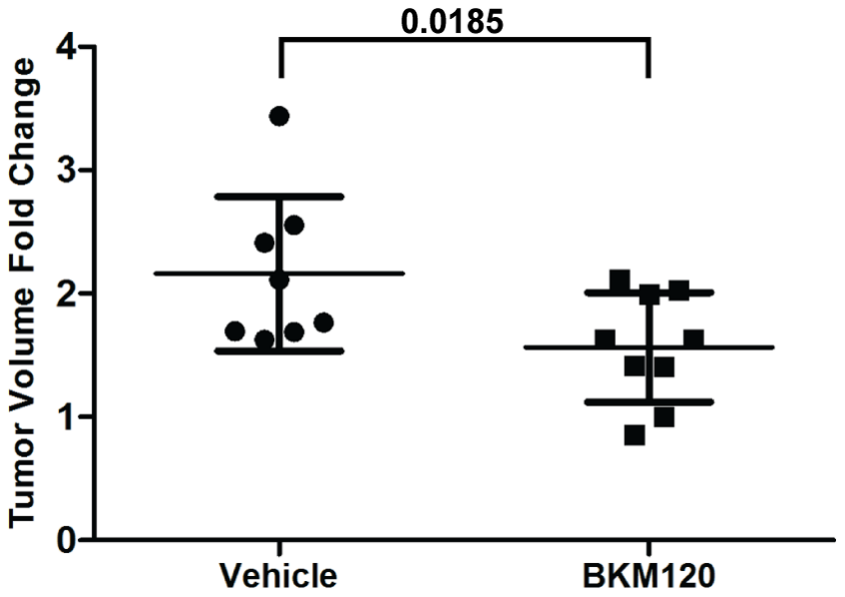
Tumor growth inhibition with BKM120 treatment. BKM120 treatment of PDAC PDX mice for 28 days inhibited tumor growth (Mean fold change, FC  = 1.56; SEM  = 0.148; n = 9) compared to vehicle (mean FC  = 2.16; SEM  = 0.221; n = 8, p = 0.0185, t-test).

We then evaluated whether this decrease in CTC burden with BKM120 treatment was associated with tumor response. Tumor volumes in the BKM120 treatment group and vehicle group were the same pre-treatment (BKM120 group: mean  = 256 mm^3^, standard deviation (SD)  = 105 mm^3^, n = 9 vs. vehicle group: mean  = 255 mm^3^, SD  = 133 mm^3^, n = 8). Mean tumor volume after 28 days of treatment in the BKM120 group was 436 mm^3^ (SD  = 270 mm^3^) compared to 540.49 mm^3^ (SD  = 301 mm^3^) in the vehicle group. BKM120 treatment inhibited tumor growth (fold change, FC  = 1.56, SEM  = 0.148) compared to vehicle (FC  = 2.16, SEM  = 0.221, p = 0.0185, t-test) over 28 days of treatment (Figure 5).

### Correlation between CTC Burden and Tumor Volume

To evaluate CTC burden as a biomarker for tumor volume, correlation between absolute CTC burden and absolute tumor volume from day 0 data was analyzed (Table 1). We found a positive but not statistically significant correlation between CTC burden and tumor volume (R = 0.558, p = 0.0547). To evaluate the change in CTC burden as a biomarker for response to therapy, we looked at the correlation between fold change in CTC burden and fold change in tumor volume over 28 days (Table 1). We found a significant correlation between the change in CTC counts and the change in tumor burden (R = 0.744, p = 0.004). However, the change in CTC count data appeared to be non-normal and the occurrence of mice with zero detected CTCs limited log transformation. These results show a stronger correlation between the change in tumor volume and the change in CTC burden than between the absolute number of CTCs and tumor size. This suggests that serially monitoring CTC burden over time to monitor response to therapy may be more clinically useful than using CTCs as a biomarker of actual tumor burden.

**Table 1:** Tumor volume and CTC burden response to treatment.

Group	Mouse ID	Tumor Volume (mm^3^)			CTC Burden (CTCs/250 µL)		
		Pre	Post	Fold Change	Pre	Post	Fold Change
Vehicle	364	147.9	239.1	1.6	42.9	10.3	0.2
Vehicle	368	158.4	267.2	1.7	9.9	6.9	0.7
Vehicle	375	126.0	304.0	2.4	36.9	12.5	0.3
Vehicle	391	239.1	405.0	1. 79	-	145.6	-
Vehicle	735	379.8	668.3	1.8	-	67.1	-
Vehicle	739	487.7	1028.5	2.1	-	11.3	-
Vehicle	740	379.3	968.0	2. 65	-	5.6	-
Vehicle	743	121.0	416.0	3.4	4.2	21.4	5.1
BKM120	361	137.3	117.0	0.9	8.8	-	-
BKM120	363	288.0	575.0	2.0	44.0	2.5	0.1
BKM120	365	147.9	208.3	1.4	20.8	3.0	0.1
BKM120	371	309.0	627.0	2.0	-	79.3	-
BKM120	387	272.0	384.8	1.4	27.5	0.2	0.0
BKM120	388	147.9	147.9	1.0	32.0	14.5	0.5
BKM120	736	210.9	343.2	1.6	25.7	1.9	0.1
BKM120	741	487.7	1028.0	2.1	63.5	0.2	0.0
BKM120	747	304.0	496.4	1.6	6.9	0	0

(-) No data due to microfluidic chip failure.

### Tumor and CTC DNA Mutation Analysis

Sequencing of DNA extracted from PDX tumor samples showed that the primary tumor was *PI3KCA* wild type at codons 542, 545, 1047 and *KRAS* G12V mutant at codon 12. To determine if CTCs were shed from the tumor MES of *KRAS* codon 12 was performed following CTC isolation. DNA was successfully extracted from 16 of 34 CTC samples. 81% (13/16) of the extracted DNA samples were of sufficient quality for mutational analysis. 100% (13/13) of CTC samples analyzed with MES had *KRAS* G12V mutations, which corresponded with the *KRAS* mutational status of matched PDX tumors, confirming capture of PDX associated CTCs.

## Conclusion

While much work has been done to prove the prognostic value of CTC levels in breast, colorectal, prostate and lung cancers, little progress has been made in this area for pancreatic cancer due to the poor sensitivity of many CTC assays for patients with PDAC [1]–[5]. Given the high sensitivity of our CTC assay for PDAC patients and the rapid deterioration of patients once they are diagnosed, a preclinical model for studying CTCs in PDAC is especially important [24]. The highly sensitive microfluidic chip used in this study was capable of detecting and enumerating CTCs from only 250 µL of PDAC PDX mouse blood. Downstream MES of isolated CTCs also showed that they harbored G12V *KRAS* mutations, as did the matched primary tumors, which is significant as it confirmed our method successfully isolated PDX derived CTCs. Cell line xenograft mice and genetically engineered mice have been used as preclinical models to study CTCs previously, but these model systems are limited by their inability to replicate the very heterogeneous population of cells found in human tumors [20], [21]. Our data suggests that PDX mice can be a useful preclinical model for studying CTCs. PDX mice harbor human tumor tissue and thus may prove to be a better model system for studying the significance of CTC burden.

Tumor growth inhibition with BKM120 was modest in this study and may be explained by the primary tumor being *PI3KCA* wild type and *KRAS* mutant. It has been shown that *KRAS* activating mutations are predictive of resistance to the PI3K inhibitor, PX-866, while *PI3KCA* mutations are predictive of sensitivity [29]. Therefore, future work with models that are *KRAS* wild type and harbor *PI3KCA* activating mutations may show a more robust response to BKM120.

Previous work in patients with epithelial based cancers showed that the absolute number of CTCs found in circulation did not correspond to tumor size. However, in a small cohort of these patients, serial measurements of CTC burden and radiographic tumor volume showed a correlation between response to treatment and no-response to treatment [30]. Similarly, prior work with breast cancer patients undergoing therapy did not find a correlation between CTC burden and radiographic evidence of tumor volume. However, the results did show that CTC burden at first follow up was a better marker for predicting survival than radiographic evidence of response [31]. In agreement with these prior studies, our results show stronger evidence for a correlation between change in tumor volume and change in CTC burden than for absolute number of CTCs correlating to tumor volume. This suggests that serially monitoring CTC burden over time to monitor response to therapy may be more clinically useful than using CTCs as a biomarker for tumor burden.

More studies will also be needed to determine whether CTCs may be utilized as an early biomarker for response to treatment before change in tumor volume is evident. To better assess this, treatment could be continued for longer than 28 days allowing time for a more pronounced response in tumor volume to be observed. A longer course of treatment would also allow for serial measurements of CTC burden over time.

In the long-term, PDX mice may be a useful preclinical model for further studying the prognostic significance of CTCs and their role in metastasis. Our results demonstrate that CTC burden may be a useful biomarker of response to targeted therapy in PDX mouse models of pancreatic cancer. These results suggest that monitoring of CTC burden in patients over the course of treatment could provide early evidence of treatment response to guide therapeutic changes and limit unnecessary prolonged exposure to toxic treatments. Investigating the mutational status of CTCs with MES could also help to identify patients that would be most responsive to mutation targeted therapy, thus serving as a minimally invasive “liquid biopsy.”
